# Debonding Characteristic and Survival Probability of Adhesive Flash-Free Ceramic Orthodontic Brackets Following pH Cycling

**DOI:** 10.1055/s-0044-1795125

**Published:** 2024-12-30

**Authors:** Tarek Ahmed Soliman, Ali Robaian, Nasser Raqe Alqhtani, Abdullah Alshehri, Abdullah Saad Alqahtahni, Ibrahim Saleh Aljulayfi, Magdy Alazzazi, Ali Elkaffas, Shahad Saleh AlGhannam, Sayed Ghorab

**Affiliations:** 1 Department of Conservative Dental Sciences, College of Dentistry, Prince Sattam Bin Abdulaziz University, Al-Kharj, Saudi Arabia; 2 Department of Oral and Maxillofacial Surgery and Diagnostic Sciences, College of Dentistry, Prince Sattam Bin Abdulaziz University, Al-Kharj, Saudi Arabia; 3 Department of Preventive Dental Sciences, College of Dentistry Prince Sattam Bin Abdulaziz University Al-Kharj, Al-Kharj, Saudi Arabia; 4 Prosthetic Dental Science Department, College of Dentistry, Prince Sattam Bin Abdulaziz University, Al-Kharj, Saudi Arabia; 5 Department of Oral Biology, Faculty of Dental Medicine, Al-Azhar University, Cairo, Egypt; 6 Oral Histology Department, College of Dentistry, Islamic University, Iraq; 7 Dental Hospital, College of Dentistry, Prince Sattam Bin Abdulaziz University, Al-Kharj, Saudi Arabia; 8 Department of Dental Biomaterials, Faculty of Dentistry, Mansoura University, Mansoura City, Egypt

**Keywords:** debonding, flash-free adhesive, pH cycling, orthodontics, survival probability

## Abstract

**Objectives** Orthodontic bracket bond failure is an obstacle in clinical orthodontics. This study investigated the influence of pH cycling on the shear bond strength (SBS), adhesive remnant index (ARI), and survival probability of adhesive-precoated flash-free ceramic brackets.

**Materials and Methods** Forty mandibular premolars were randomly divided into two groups ( *n* = 20): C: noncoated orthodontic brackets, and F: flash-free adhesive-precoated orthodontic brackets. Each group was subdivided into two subgroups according to storage medium solutions ( *n* = 10): in subgroup AS, specimens were immersed in artificial saliva for 24 hours, and in Subgroup ASL, specimens were recycled between a demineralizing solution and an artificial saliva for 42 days. Within each subgroup, specimens were subjected to SBS and ARI testing. SBS data were analyzed using one-way ANOVA (analysis of variance) and Tukey's post-hoc test. Weibull analysis was performed on the SBS data to determine the characteristic SBS and their survival probabilities.

**Results** Flash-free adhesive-precoated brackets had higher significant ( *p* < 0.001) SBS values in both the AS group (17.74 ± 1.74 MPa) and the ASL group (12.61 ± 1.40 MPa) compared with the noncoated bracket (10.67 ± 1.55 and 7.89 ± 1.39 MPa, respectively). The ARI scores for the noncoated brackets in the AS group were 70% occurrence for score 1, while 90% for score 1 in the ASL group. For the flash-free precoated brackets, ARI scores were 70% occurrence for score 2 in the AS group, while 80% for score 2 in the ASL group. Flash-free brackets had higher SBS in both AS and ASL groups (14.07 and 9.76 MPa, respectively), at 95% survival probability.

**Conclusion** Flash-free orthodontic brackets performed better in terms of significantly higher bond strength and higher ARI scores. Meanwhile, noncoated brackets revealed acceptable SBS results in both storage medium groups. Flash-free brackets showed higher survival than the noncoated brackets in both storage medium groups at 90% survival probability.

## Introduction

White spot lesion (WSL) formation is a prevalent and significant problem in orthodontics. 1 2 3 The presence of fixed orthodontic appliances with their irregular surfaces, combined with variations in the oral environment and excessive adhesive, contributes to plaque accumulation around brackets and increases the level of cariogenic bacteria. 4 5 The acids produced by these bacteria result in demineralization of the enamel surface, 6 compromising the retention of brackets 7 and raising aesthetic concerns during fixed orthodontic treatment. 8

The bond strength between the bracket and the enamel is critical to the success of orthodontic treatment because it ensures that the orthodontic forces are applied correctly. To achieve a positive outcome, measures must be taken to prevent the formation of carious lesions around and beneath orthodontic appliances. 9 10 The prevalence of enamel demineralization in orthodontic patients due to bacterial acid was found to be higher than 45%. Surface roughness caused by remaining adhesive residue around brackets contributes to this type of demineralization, which eventually leads to plaque accumulation. 10 11

The ideal orthodontic adhesive should preserve the enamel's integrity, provide a good seal with a minimum excess adhesive around the margins, and offer sufficient bond strength to prevent bracket detachment during the tooth movement. 11 These features help oral hygiene maintenance and avoid the development of rough surfaces that could consequently provide effective bonding. The increased accumulation of plaque in the oral environment triggers the development of WSLs, which is the main contributing factor to bonding failure. 12

Most orthodontists manually apply orthodontic adhesive to the bracket bases prior to their insertion. Although it is better to remove the flash completely, orthodontists sometimes leave it in place, exposing a rough composite surface, which can contribute to the accumulation of plaque, enamel demineralization, and the formation of white spotlesions. 11 12 13 A flash-free adhesive-precoated bracket system has been introduced with the objective of minimizing the removal of excess adhesive, improving the bond strength, and ensuring a smooth margin at the bracket/adhesive interface. These improvements aim to minimize plaque accumulation and the formation of WSLs. 14 15 16 17 18

The adhesive-precoated system comprises a ceramic bracket with low-viscosity resin attached to its base. Eliminating resin flash is unnecessary with this system, thus preventing the development of rough surfaces and plaque buildup, which could hinder effective adhesion. Although flash-free adhesive brackets have demonstrated satisfactory results with regard to bond strength and chair time, 18 19 further *in vitro* studies in clinically relevant regimens are needed. This is crucial as pH fluctuations in the oral environment can potentially cause mineral loss in enamel, posing a challenge for adhesion.

Therefore, the objective of this study was to investigate the influence of pH cycling on the shear bond strength (SBS), adhesive remnant index (ARI), and the survival probability of adhesive-precoated flash-free ceramic brackets. The null hypotheses stated that there would be no statistically significant differences between flash-free adhesive-precoated ceramic brackets and the traditional noncoated ceramic brackets following pH cycling with regard to (1) SBS, (2) ARI, and (3) survival probabilities.

## Materials and Methods

### Specimen Selection

#### Sample Size Calculation

A power analysis was conducted utilizing G* Power computer software (version 3.1.9.2, Heinrich Heine Universität, Düsseldorf, Germany) to determine the appropriate sample size. A sample size of 40 specimens (10 per group) was necessary to achieve a power of 0.95 at 0.05 significance level with an estimated size effect of 2.34.

#### Specimens' Inclusion Criteria

Forty mandibular premolars were obtained from orthodontic patients undergoing extractions at the Faculty of Dentistry, Mansoura University's Orthodontic Outpatient Clinic. These premolars had intact buccal enamel and were carefully selected based on their soundness, no evident enamel damages, filling or carious lesions, lack of fractures, cracks, and demineralization. The selected teeth were screened using an optical microscope. The extracted premolars were preserved in 0.1% thymol solution for 24 hours to inhibit bacterial growth. To ensure cleanliness, the premolars were cleansed using a pumice slurry in a rubber prophylactic cup at low speed. They were then stored in distilled water at room temperature, with regular water changes to prevent dehydration. This study received approval from the ethics committee at Mansoura University's Faculty of Dentistry, under reference number M0106023DM.

### Study Design and Specimens' Grouping

The premolars were randomly divided into two groups ( *n* = 20) according to the bracket/adhesive system used: C: noncoated ceramic brackets with adhesive at the time of bonding (Clarity Advanced ceramic brackets), and F: adhesive-precoated brackets (APC Flash-Free). The materials used in the study are presented in Table 1 . Each group was subdivided into two subgroups ( *n* = 10) according to the demineralization storage media: Subgroup 1 (AS):specimens were submerged in artificial saliva for 24 hours, and Subgroup 2 (ASL): specimens were recycled between the artificial cariogenic solution and an artificial saliva for 42 days (6 weeks) to simulate artificial caries like lesion. 16

**Table 1:** Materials used in the study

Product	Composition/manufacturer	Indication	Lot. No.
APC Flash-free adhesive coated orthodontic ceramic brackets	- Precoated ceramic brackets - A unique low viscosity, nano-filled methacrylate-based resin with compressible nonwoven polypropylene fibers/3M Unitek (Monrovia, California, United States)	Orthodontic treatment brackets	HU5ZX
Clarity Advanced Ceramic brackets	-Noncoated ceramic orthodontic brackets/3M Unitek (Monrovia, California, United States)	Orthodontic treatment bracket	JV6QR

### Bracket Bonding Procedure

Following the manufacturer's instructions, the buccal enamel surface of each premolar was conditioned using Transbond Plus self-etching primer. The ceramic brackets were bonded to the conditioned surfaces using the adhesive systems. The adhesive-precoated ceramic brackets were carefully placed on the surface of the specimens and adjusted to their final position. A standardized constant force was applied to ensure a consistent adhesive thickness on the top surface of the brackets. For the group using traditional noncoated brackets, Transbond Plus Self-Etch Primer (3M Unitek, Monrovia, California, United States) was applied on the enamel surfaces, and Transbond XT composite (3M Unitek, Monrovia, California, United States) was used to adhere the brackets. Any excess adhesive was removed using an explorer. The adhesive resin was polymerized using the curing light (3M Unitek; Monrovia, California, United States; light output: 1,600 mW/cm ^2^ ) for 12 seconds from two different directions. 20 21 Subsequently, the specimens were placed in distilled water at 37°C for 24 hours to ensure complete polymerization.

### Storage Media Immersion Protocol

In the AS subgroup, the specimens were placed in artificial saliva containing 20 mmol/L NaHCO _3_ , 3 mmol/L NaH2PO _4_ , and 1 mmol/L CaCl _2_ at a neutral pH for 24 hours. 16 For the ASL subgroup, the specimens alternated between two solutions. They were immersed in 5 mL of the demineralizing solution for 20 minutes three times a day, with intervals in artificial saliva. The demineralization solution consisted of 3.0 mmol/L CaCl _2_ , 1.8 mmol/L KH2PO _4_ , 0.1 mol/L lactic acid, and 1% carboxymethylcellulose, with pH adjusted to 4 using KOH. 20 22 Following acidic exposure, the specimens were rinsed with distilled water to eliminate any acidic residue. The pH cycling procedure was performed at room temperature and repeated daily for 42 days. 20 22 The solution was changed daily to maintain a consistent pH of 4.

### Shear Bond Strength Test and Adhesive Remnant Index

An Instron universal testing machine (AGS-1000A; Shimadzu Co., Kyoto, Japan) was used to conduct SBS testing. The specimens were securely clamped in the lower jaw of the machine, ensuring alignment with the shear force's direction. A stainless steel mono-beveled chisel was accurately positioned on the bracket base. Then, a compressive force with a crosshead speed of 0.5 mm/min was applied. 23 24 The shear force at fracture was recorded using a 2.5 kN load cell linked to a computer, with the results presented in Newtons (N) by the testing machine. 21 The SBS (MPa) is calculated by dividing the fracture load ( *F* ) in Newtons by the surface area ( *A* ) in mm ^2^ . Following debonding, the fractured specimens were examined for the remaining adhesive using an optical stereomicroscope (Olympus SZ61, Tokyo, Japan) at a 40× magnification level. The evaluation was performed using the subsequent criteria 25 : 0: absence of adhesive on enamel; 1: less than half of the adhesive on enamel; 2: more than half of the adhesive on enamel; 3: all adhesive on enamel. The ARI scores were utilized to identify the failure sites between the enamel, adhesive resin, and bracket base.

### Statistical Analysis

SBS data were analyzed using one-way ANOVA (analysis of variance) to detect significant differences among groups. Tukey's significant difference tests were used for post-hoc comparisons. Furthermore, two-way ANOVA was applied to detect the interactions between the independent variables (bracket type and demineralization). The Independent sample *t* -test was used to compare SBS values between the two orthodontic brackets in each storage media. The Chi-square (χ ^2^ *)* test was used to determine significant differences ( *p* < 0.05) in the ARI scores.

The SBS data were analyzed using the Weibull analysis to calculate the SBS at different survival probabilities ( *P*_s_ ). The Weibull distribution formula is expressed as *P*_s_ = EXP [−(σ/σ0) *m* ], where *P*_s_ represents the survival probability at any given shear stress, σ denotes the SBS at any given stress, σ0 stands for the characteristic bond strength, and *m* is the Weibull modulus.

## Results

The normality of SBS data and equal variance assumption were fulfilled according to the Shapiro–Wilk test ( *p* > 0.05) and Levene's test. The means and standard deviations of SBS values (MPa) for all groups are presented in Table 2 . The flash-free bracket system had higher significant ( *p <* 0.001) SBS values in both the AS group (17.74 ± 1.74 MPa) and the ASL group (12.61 ± 1.40 MPa) compared with the noncoated ceramic bracket (10.67 ± 1.55 MPa and 7.89 ± 1.39 MPa, respectively). Furthermore, the overall SBS values of flash-free orthodontic brackets were significantly higher ( *p <* 0.001) (15.18 ± 3.04 MPa) than the noncoated ceramic bracket (9.28 ± 2.02 MPa). A paired *t* -test showed that the demineralization storage solution significantly reduced ( *p <* 0.001) SBS values for both flash-free orthodontic brackets and the noncoated ceramic bracket ( Table 2 ). The two-way ANOVA table ( Table 3 ) shows that the independent variables (bracket type and demineralization) have a significant effect on SBS data.

**Table 2:** Shear bond strength (SBS) in MPa for the different groups

Storage media groups	Orthodontic brackets		Overall SBS
	Noncoated bracket (C)	Flash-free bracket (F)	
AS	10.67 ± 1.55 ^A,b^	17.74 ± 1.74 ^A,a^	14.20 ± 3.96 ^A^
ASL	7.89 ± 1.39 ^B,b^	12.61 ± 1.40 ^B,a^	10.25 ± 2.77 ^B^
Overall SBS	9.28 ± 2.02 ^b^	15.18 ± 3.04 ^a^	

Abbreviations: AS, specimens immersed in artificial saliva; ASL, specimens were recycled between the artificial cariogenic solution and an artificial saliva.

Note: Mean values represented with different superscript lowercase letters (row) are significantly different according to Tukey's significant different test ( *p <* 0.05) *.* Mean values represented with different superscript uppercase letters (column) are significantly different according to Tukey's significant different test ( *p <* 0.05) *.*

**Table 3:** Two-way ANOVA table for shear bond strength (MPa)

Source of variations	Sum of squares	*df*	Mean squares	*F*	*p-* Value
Type of bracket	347.45	1	347.45	148.92	*p <* 0.001
Demineralization	156.14	1	156.14	66.92	*p <* 0.001
Type of bracket × demineralization	13.81	1	13.81	5.92	*p = 0.02*
Corrected total	601.406 40				

Abbreviation: ANOVA, analysis of variance.

**Fig. 1:**
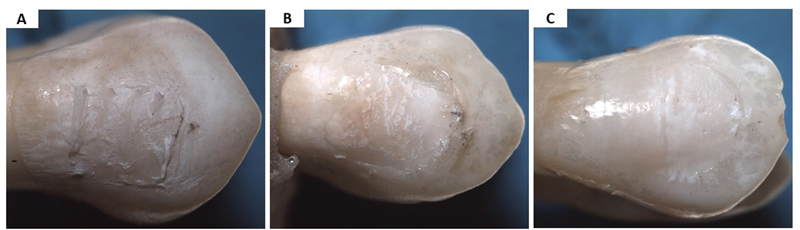
ARI scores for different groups. ( **A** ) Remnant adhesive represents score 2; ( **B** ) remnant adhesive represents score 1; ( **C** ) ARI score 0. ARI, adhesive remnant index.

The brackets' adhesive type (χ ^2^ = 28.8, *p <* 0.001) and the storage media solution (Monte Carlo test, *p* < 0.001) significantly affect ARI scores. The percentage of ARI scores is presented in Table 4 . The percentage of ARI scores for the noncoated brackets in the AS group was 70% for score 1, while 90% for score 1 in the ASL group. For the flash-free precoated brackets, the percentage of ARI scores was 70% occurrence for score 2 in the AS group, while 80% occurrence for score 2 in the ASL group. Fig. 1 shows the different score occurrences in the tested groups representing scores 0, 1, and 2.

**Table 4:** Frequency distribution percentages of adhesive remanent index (ARI) scores for different groups

ARI scores	Noncoated brackets		Precoated flash-free brackets	
	AS	ASL	AS	ASL
0	0%	0%	0%	10%
1	70%	90%	30%	10%
2	30%	10%	70%	80%
3	0%	0%	0%	0%

Note: 0: absence of adhesive on enamel; 1: less than 50% of adhesive on enamel; 2: more than 50% of adhesive on enamel; 3: all adhesive on enamel.

**Fig. 2:**
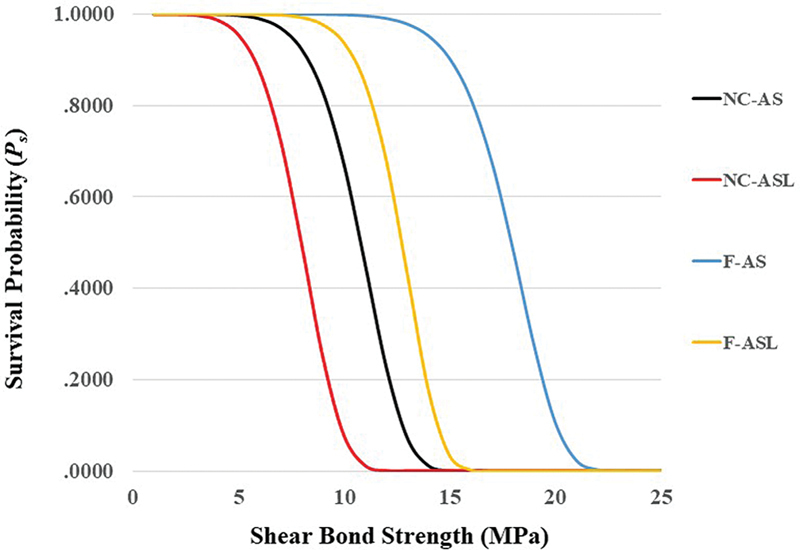
Weibull survival probability curves for different groups.

Table 5 displays the Weibull parameters for each group. The Weibull modulus values for each material varied with each storage medium solution and the bracket type, showing higher values for the flash-free brackets in AS and ASL groups. The survival probability plot curves for the different groups are presented in Fig. 2 .

**Table 5:** Weibull analysis of SBS data in all groups

	Weibull parameters					
Type of bracket/storage media	*m*	*r*	σ _0_	SBS at *P*_s_		
				5%	90%	95%
**Noncoated brackets**						
AS	7.19	0.89	11.36	13.24	8.31	7.52
ASL	5.80	0.98	8.51	10.28	5.77	5.09
**Flash-free bracket**						
AS	10.75	0.81	18.55	20.54	15.04	14.07
ASL	9.77	0.96	13.23	14.80	10.51	9.76

Abbreviations: AS, artificial saliva group; ASL; cyclic immersion between artificial saliva and lactic acid group; *m,* Weibull modulus; *r,* correlation co-efficient; σ _0_ , characteristic bond strength; SBS, shear bond strength; *P*_***s***_ , survival probability.

## Discussion

During orthodontic treatment, it is crucial to maintain the bond between the bracket and the enamel to ensure accurate application of force. Therefore, preventing demineralization around orthodontic appliances is essential for both the patient's oral health and the successful completion of a lengthy and costly orthodontic treatment. 26 27 28 Specimens were recycled three times a day between artificial saliva and lactic acid for 42 days to simulate the cariogenic environment that adhesive–enamel-bonded areas would encounter *in vivo* . It is worth noting that demineralization could potentially occur as soon as 1 month after the placement of brackets. 22 29 30 Lactic acid, with a pH of 4, was chosen as the demineralizing solution because it is the primary acid produced by plaque microorganisms. 4 16 The three-times-per-day interval was selected to stimulate the pH conditions experienced in the oral environment during consumption of fermentable food three times a day, i.e., three meal periods. 31 Accordingly, the present study was conducted to investigate the influence of pH cycling on the SBS, ARI, and the survival probability of adhesive-precoated flash-free ceramic brackets.

Mandibular premolars were chosen due to their significant rate (61%) of bracket failures. 19 Although metal brackets showed higher bond strength than ceramic brackets, according to a previous study, ceramic brackets recorded SBS beyond the clinically acceptable value for bracket bond strength. 14 32 In this study, two types of ceramic orthodontic brackets were utilized for standardization purposes: one that required the removal of resin flash (Advanced Clarity) and another that did not (flash-free adhesive). 14 15 17 In this study, the self-etching adhesive was utilized due to its ability to decrease clinical application time, enhance cost-effectiveness, and improve patient comfort. While the conventional etch and rinse adhesive delivers the best bond strength, it involves several time-consuming steps. 33 Furthermore, a previous study reported that, although self-etching adhesives showed the lowest bond strengths, they caused limited enamel damage and generally left less residual resin on the tooth structure. 34 The shear test, which is widely used in laboratory settings, was employed to assess the SBS of the brackets. A crosshead speed of 0.5 mm/min was selected for this test as it is less affected by handling and allows for an even distribution of stress at the bonded interface. Although debonding forces are more likely to affect the bracket wings *in vivo* , the shear wedge was carefully positioned on the bracket base to prevent rotational stresses. 35 36

According to the findings of this study, the flash-free orthodontic bracket group had a significantly higher overall SBS (15.18 ± 3.04) than the noncoated orthodontic brackets (9.28 ± 2.02). This might be explained by the unique resin characteristic utilized in flash-free adhesives. It is a low-viscosity adhesive resin that can easily wet and penetrate surfaces, thereby improving wettability and adhesion. 17 Furthermore, achieving a more uniform distribution of adhesive between the bracket base and the enamel could reduce resin flashes surrounding orthodontic brackets and minimize changes to the enamel surface. The excess adhesive at the bracket margins allows bacteria to accumulate, resulting in increased demineralization and reduced bond strength in traditional noncoated brackets. 15 16

The findings of this study are in line with other similar studies that reported that flash-free adhesives recorded significantly higher SBS than conventional noncoated ceramic brackets. 14 19 21 37 38 .

Conversely, another study concluded that flash-free and conventional adhesive ceramic brackets demonstrated comparable clinical performances, with the exception of the quicker bonding achieved with the flash-free adhesive. 39 On the other hand, the SBS of the noncoated orthodontic bracket was significantly ( *p* < 0.05) reduced due to the effect of demineralizing storage medium solution. Consequently, the first null hypothesis was rejected.

Reynold 40 mentioned that 5.9 to 7.8 MPa is a sufficient bond strength for the orthodontic brackets to withstand orthodontic forces applied during treatment. In the current study, all groups had bond strength values greater than 6 MPa, which could be considered adequate for clinical applications. On the other hand, Bishara et al 41 and Lee and Kanavakis 14 reported that 10 MPa is the appropriate bond strength for orthodontic purposes. As a result, all groups had bond strength values greater than 10 MPa except the noncoated brackets in the ASL group.

A key consideration in choosing orthodontic adhesives is the amount of residual adhesive after debonding, which can be measured using the ARI scoring system. The ARI score was utilized to identify the location of deboned interface failure during SBS testing. 20 The type of brackets and the storage medium solution significantly affect ( *p <* 0.001) ARI scores. Accordingly, the second null hypothesis was rejected. The occurrence of bracket failures using flash-free adhesives was predominantly observed in score 2, which are considered more advantageous in preventing enamel fractures during the debonding process. The higher ARI score may be due to the compressible nonwoven polypropylene fiber attached to the bracket base to retain excess adhesive squeezed out during bracket application. 42 43

The mean SBS values do not accurately represent the true strength values, which can lead to misinterpretation. This could explain the discrepancy between our study's mean bond strength and the Weibull analysis findings. 44 45 Weibull analysis is recognized as a reliable method for assessing fracture behavior by focusing on data distribution instead of mean average values, thus confirming the trustworthiness of laboratory data for clinical usage. Additionally, data indicating a survival probability exceeding 90% with reliable bond strength were obtained. Furthermore, predicting material failure at any relevant stress level is also achievable. 45 46

Considering the clinically acceptable value for bracket bond strength (10 MPa; 14.35), it is possible to conclude that flash-free brackets showed satisfactory performance because stress values exceeded 10 MPa in both the AS (15.04 MPa) and ASL (10.51 MPa) groups at 90% survival probabilities. Regarding 95% survival probabilities, flash-free brackets stress values exceeded 10 MPa in the AS group only (14.07 MPa). Accordingly, the third null hypothesis was partially accepted.

The orthodontic field is constantly evolving in terms of techniques and materials, with the goal of developing more efficient and effective patient treatment methods. The ability to remove brackets after treatment without damaging the underlying enamel is of particular concern to orthodontists. When compared with noncoated brackets, flash-free orthodontic brackets performed better due to higher bond strength, lower ARI scores, and higher survival probability values. The study's findings can assist orthodontists in determining which orthodontic appliances are best for their practice. The orthodontist decides whether to use noncoated or flash-free orthodontic brackets, but both must meet the needs of the practice, particularly in terms of bond strength, ARI scores, and survival probabilities.

The study's limitations lie in its lack of accurately replicating the real-life oral conditions. *In vivo* , the forces exerted on orthodontic brackets are multifaceted, encompassing shear, tensile, and compressive stresses. Future studies should investigate the attachment of bacteria to flash-free precoated orthodontic brackets.

## Conclusion

Flash-free orthodontic brackets performed better in terms of significantly higher bond strength and lesser ARI score. Meanwhile, noncoated ceramic brackets revealed acceptable SBS results in both storage medium groups. Flash-free brackets showed higher survival than the noncoated brackets in both storage medium groups at 90% survival probability.
